# Effects of multicomponent exercise nursing intervention in elderly stroke patients with frailty: a randomized controlled trial

**DOI:** 10.3389/fmed.2024.1450494

**Published:** 2024-10-02

**Authors:** Yanfang Luo, Jianru Hao, Lingyun Zhu, Yujuan Huang, Zhimin Liu, Yuping Chen, Yuyu Qiu, Zhenzhen Su, Renjuan Sun

**Affiliations:** ^1^Department of Neurology, Affiliated Hospital of Jiangnan University, Wuxi, China; ^2^Wuxi School of Medicine, Jiangnan University, Wuxi, China; ^3^Department of Basic Medicine, Jiangsu Vocational College of Medicine, Yancheng, China

**Keywords:** elderly, stroke, frailty, multicomponent exercise, nursing intervention, activities of daily living, quality of life

## Abstract

This study examines how multicomponent exercise nursing interventions affect the state of frailty, daily activities, and quality of life in elderly stroke patients with frailty. A total of 125 elderly stroke patients with frailty were randomly assigned to either a control group (*n* = 62) or an intervention group (*n* = 63). The control group received standard nursing care, while the intervention group received a multicomponent exercise nursing intervention in addition to standard care. Patients were assessed using the FRAIL Frailty Scale, Modified Barthel Index (MBI), and Short Form Health Survey (SF-36) before the intervention, 4 weeks after the intervention, and 12 weeks after the intervention. Significant differences were observed between the two groups in terms of frailty status, activities of daily living, and quality of life (*p* < 0.05). The intervention group had lower scores on the FRAIL Frailty Scale and higher scores on the MBI and SF-36 compared to the control group at both 4 and 12 weeks after the intervention (*p* < 0.05). These findings suggest that multicomponent exercise nursing interventions can effectively reduce frailty and improve activities of daily living and quality of life in elderly stroke patients with frailty.

## 1 Introduction

Frailty is characterized by a decline in physical function and increased vulnerability to imbalances after a stressful event (1). Frail individuals require comprehensive healthcare interventions, as they are at a higher risk for negative health outcomes like dependency and disability (2). This is especially common among older adults, who are more likely to experience accelerated decline in physical and cognitive abilities and have higher mortality rates (3). A systematic review and meta-analysis encompassing 1,187,000 individuals with stroke in 2022 revealed a frailty prevalence of 39.7% (4). Frailty can lead to a decline in motor function among patients (5). This deterioration in motor function can directly impair their ability to perform activities of daily living and subsequently affect their overall quality of life. Evidence indicates that the quality of life for patients experiencing frailty post-stroke is markedly lower compared to those without frailty, with a more pronounced decline observed over time (6). Frail stroke individuals also have a higher risk of short and long-term mortality. Research has shown that frailty is a significant predictor of increased mortality within 3 months after a stroke (7). In addition, the patient’s ability to perform activities of daily living is also an important issue for individuals with stroke. A systematic review demonstrated a robust correlation between stroke and activities of daily living, providing substantial evidence that stroke significantly impairs daily functioning in elderly individuals (8). This impairment underscores the critical importance of restoring physical activity in individuals with stroke. Therefore, it is crucial to implement appropriate treatment strategies to mitigate frailty in elderly individuals with stroke, improve activities of daily living, and minimize its adverse effects on health outcomes.

Non-pharmacological interventions are strategies that do not involve medication and are used for preventing, managing, or treating diseases and health conditions. These interventions aim to improve patients’ quality of life, enhance functional abilities, and alleviate symptoms (9). Research suggests that non-pharmacological interventions, particularly exercise interventions, are effective in addressing frailty in the elderly (10). Implementing exercise interventions has been shown to improve frailty status, enhance daily activities, reduce anxiety and depression levels, and alleviate other negative emotional states (11). The 2018 International Conference on Skeletal Muscle Reduction Disorder and Frail Research (ICSFR) recommended the use of progressive multicomponent exercise interventions for frail older adults to promote healthy aging (12).

A comprehensive exercise program called multicomponent exercise, which includes flexibility, balance, coordination, resistance, and aerobic training, has been proven to improve muscle strength, balance, flexibility, and walking speed. This leads to better health outcomes for patients (13). Multicomponent exercise interventions have demonstrated clinical significance in a variety of diseases. A 12-month intervention study conducted in a Spanish community showed that a progressive and personalized multicomponent exercise program effectively reduced falls, improved frailty, and decreased mortality rates among older adults (14). Another cohort study on older adults recovering from COVID-19 in intensive care found that multicomponent exercise interventions significantly improved frailty, balance, gait, and muscle strength in this population (15). Additionally, multicomponent exercise is highly applicable to the elderly population. A study targeting elderly individuals in the community has demonstrated that multicomponent exercise can significantly improve frailty status and activity levels in the elderly (16). Multiple studies have indicated that multicomponent exercise can notably enhance the quality of life for older adults (17–19). In addition, the effects of the exercise are long-lasting. A cohort study in elderly individuals with diabetes showed a significant effect of a multicomponent exercise intervention (20). This suggests that the exercise has a profound effect on the health of patients. These interventions come in various forms and are easy to learn and implement with minimal limitations. Multicomponent exercise is widely recognized as the most effective approach to addressing frailty globally (12).

Nursing theories play a crucial role in guiding interventions and helping healthcare providers deliver patient-centered care. These theories ensure consistency and replicability across different settings, leading to a reduction in unnecessary medical procedures and costs. This results in more efficient resource utilization, improved health outcomes, and enhanced patient satisfaction (21). The PRECEDE-PROCEED model, based on health belief theory, comprehensively analyzes and predicts individual health behaviors, facilitating the development of impactful health education and promotion initiatives. This model, proposed by psychologist Irwin M. Green in the 1950s, has gained significant recognition in the fields of public health and behavioral sciences (22). Watson’s humanistic Care model, developed by John Paul Watson, a philosopher and psychologist from Fordham University, emphasizes the importance of human emotions, relationships, and compassionate understanding in ethical conduct and decision-making. In this model, caregivers are driven by a genuine concern for the well-being and needs of others, rather than solely by duty or obligation. This model has been widely used in nursing interventions and has yielded notable results (23).

Although multicomponent exercise has shown significant effects on enhancing frailty, there is currently limited research on older adults who have had a stroke. In addition, many existing interventions lack theoretical foundations and fail to incorporate important motivational factors and compassionate care. This study aimed to develop a multicomponent exercise nursing intervention for elderly stroke patients with frailty, integrating elements of the PRECEDE-PROCEED model and Watson’s caring theory. A randomized controlled trial was conducted to examine how multicomponent exercise nursing interventions affect the state of frailty, daily activities, and quality of life in elderly stroke patients with frailty. The findings of this study aim to provide new insights in the field of rehabilitation for elderly individuals with stroke.

## 2 Materials and methods

### 2.1 Study design

This single-blind, randomized controlled trial was conducted over a period of 3 months. The study received approval from the Research Ethics Committee of the Affiliated Hospital of Jiangnan University (LS2023064) and was registered at www.chictr.org.cn. Written informed consent was obtained from all participants. After baseline measurements were taken, the participants were randomly assigned to either the intervention (IG) or control (CG) groups using a random number table.

### 2.2 Participants

**Figure 1:**
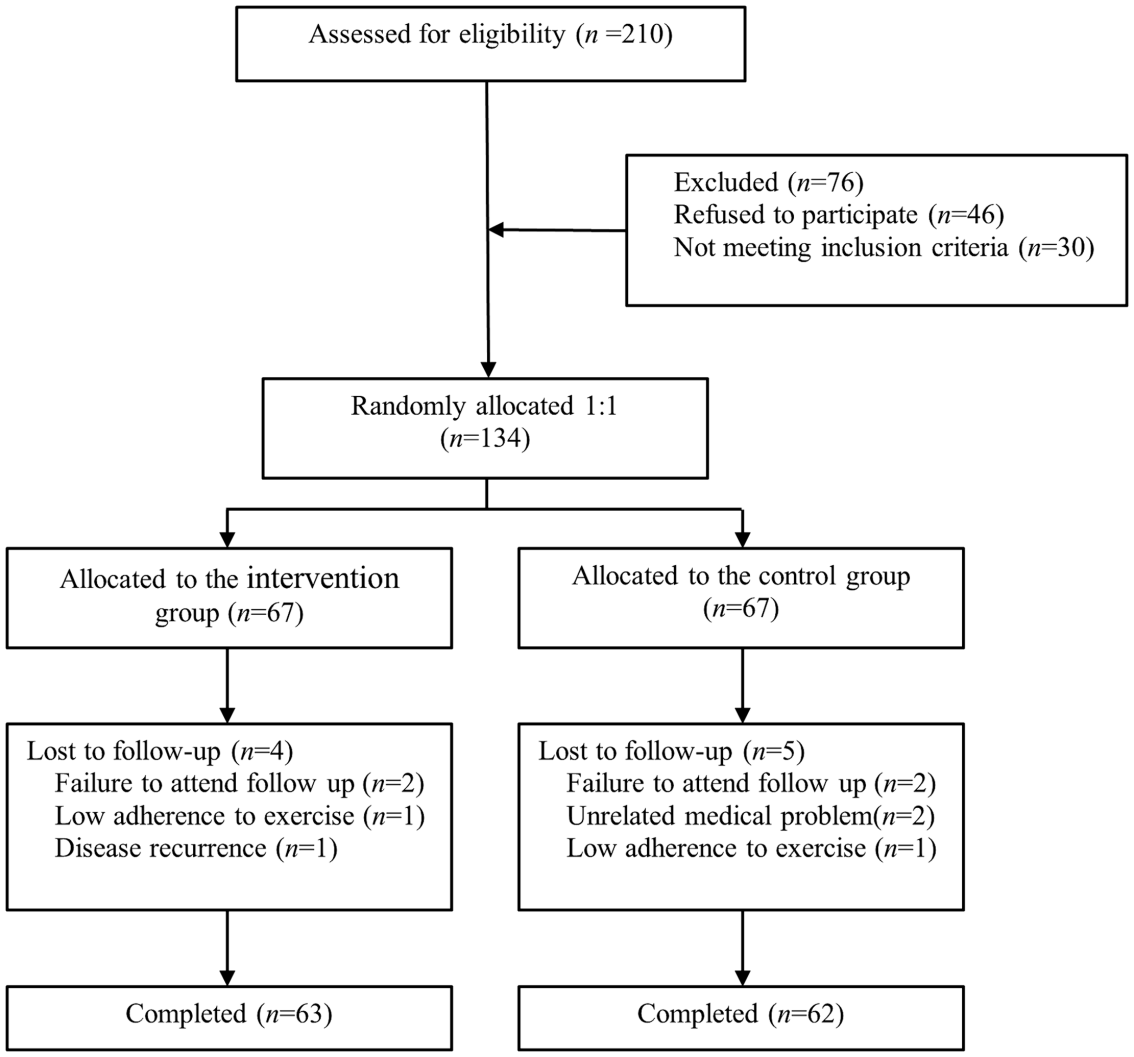
Flow chart of participants’ enrollment.

A total of 125 elderly stroke patients with frailty were recruited from a tertiary hospital in Wuxi city (Figure 1). The inclusion criteria were as follows: (1) patients who met the diagnostic criteria for acute ischemic stroke and were confirmed by head CT or MRI; (2) first episode, stable, and in the convalescent stage; (3) age ≥ 60 years; (4) FRAIL score ≥ 3; (5) bilateral upper and lower limb muscle strength ≥4 on the Lovett scale, and able to safely complete the 6-min walk test without assistance; (6) no physical or occupational therapy; (7) patients or caregivers proficient in operating smartphones; and (8) subjects voluntarily participated in the study and signed the informed consent. The exclusion criteria were: (1) accompanied by disturbance of consciousness or mental illness; (2) severe aphasia or communication disorder; (3) severe heart, lung, liver, kidney diseases, or malignant tumors; and (4) legal blindness or severe visual impairment.

### 2.3 Control group

The control group was administered standard neurological nursing care encompassing education on stroke-related conditions, medication effects, dietary and sleep recommendations, and the importance of exercise in stroke rehabilitation. Before discharge, patients received a comprehensive instructional video detailing exercise guidance. Post-discharge, patients participated in biweekly telephone follow-ups, during which nurses provided routine health education. This included highlighting the significance of smoking cessation, alcohol moderation, maintaining regular sleep patterns, and engaging in consistent physical activity for the prevention of recurrent strokes. Patients were also educated on self-monitoring techniques for essential physiological parameters such as blood pressure and blood glucose levels, and were encouraged to document their daily activity levels and dietary habits. Furthermore, strategies for emotional recognition and management were imparted to the patients.

### 2.4 Intervention group

A multicomponent exercise nursing intervention, developed through a literature review and Expert Panel Meeting, incorporated humanistic care, health education, and tailored exercise prescriptions. Grounded in Watson’s Humanistic Care model, the program focused on patient and family needs, integrating care into daily treatment while monitoring psychological and emotional well-being. The protocol included trust-building and a comprehensive assessment of patients’ psychological state, disease understanding, social support, routines, and exercise habits. Biweekly follow-up calls ensured adherence, addressed issues, and provided support. Health education, based on the PRECEDE-PROCEED model, aimed to modify behaviors and improve outcomes. On-site training guided patients and families in multicomponent exercises, with details on exercise suitability, frequency, and intensity. Post-discharge, regular follow-ups via outpatient services, phone consultations, and WeChat offered continued guidance and support (Table 1).

**Table 1:** Multicomponent exercise prescription for elderly stroke patients with frailty.

Forms of exercise	Content of exercise	Phase 1	Phase 2	Phase 3	Phase 4
		1–2 weeks after discharge	3–4 weeks after discharge	5–8 weeks after discharge	9–12 weeks after discharge
Warm up	Neck exercise: gently spin the head in a clockwise direction and then counterclockwise direction. Shoulder exercise: slowly rotate the shoulders in a back-and-forth and rotational movement. Arm and wrist exercise: gently bend and straighten the arms while rotating the wrists. Leg exercise: perform leg extension and rotational movements. Ankle exercise: perform a gentle rotational motion of the ankles, moving them back and forth and from side to side.	5–10 min each time, RPE* 13–14	10–15 min each time, RPE 13–15	As before	As before
Aerobics	Walking or jogging	5–10 min each time, RPE 13 to 15	10–15 min each time, RPE 13–15	As before	As before
Daily living skills training	Walking and stair training. Housework training: sweeping the floor, wiping the table, washing dishes, and other simple housework activities.	Walking and stair training, 5–10 min/time, housework training ≥1 item/day, RPE 13–15	Walking and stair training, 10–15 min/time, housework training ≥2 item/day, RPE 13–15	As before	As before
Elastic band resistance training	The elastic band is knotted and fixed at the height of the subject’s ankle, the subject stands facing the elastic band and can maintain balance with the help of a support. The subject stands on one leg and extends one leg backward, keeping the knee joint unbent for 15 s, alternating between the two legs.	5–10 min each time, RPE 13–15	10–15 min each time, RPE 13–15	As before	As before
Balance training	Sit with a Bobath handshake and try to stretch in all directions. Gradually transition from double bridge to single bridge in supine position.	5–10 min each time, RPE 13–15	10–15 min each time, RPE 13–15	As before	As before
Flexibility training	Static stretching training: the patient was placed in the supine position and stretched the upper and lower limbs	5–10 min each time, RPE 13–14	10–15 min each time, RPE 13–15	As before	As before

*RPE: the Rating of Perceived Exertion.

Seventy-two hours before discharge, patients and their families received instructions on a multicomponent exercise intervention, including specific movements, key safety points, and emergency protocols. A WeChat group was created to share exercise videos for review and practice. Family members were tasked with supervising the exercises and documenting attendance with photos at the start and end of each session. The study implemented a 12-week intervention cycle, and the program we implemented was as follows: week 1 to week 2—intervention once a week, week 3 to week 4—intervention twice a week, week 5 to week 8—intervention five times a week, week 9 to week 12—intervention six times a week. Patient vital signs were monitored before and after the intervention, with biweekly follow-ups conducted either online or offline. Exercise intensity was evaluated based on the patients’ subjective feelings during exercise, and the target intensity was determined using the Rating of Perceived Exertion (RPE). Based on our inclusion and exclusion criteria, we conducted preliminary experiments with six participants. The results indicated that the patients perceived the exercise intervention as causing a fatigue level of 13–15 on the 20-point RPE Borg scale (24). Therefore, we set the exercise intensity within this range to better match the exercise profile of this population. Patients were instructed to exercise within the target intensity range. We instructed patients to rest independently for mild discomfort and promptly alert researchers for symptoms such as paroxysmal chest tightness, palpitations, muscle weakness, headache, and other serious discomfort.

### 2.5 Outcomes measures

Data were collected at three time points: baseline, week 4, and week 12. This study employed a combination of on-site surveys and online follow-up. Researchers were trained in standardized instruction language for surveying participants. The study objectives, training procedures, and key points were clearly communicated to the researchers. Individuals with stroke independently and anonymously completed questionnaires during on-site visits, which were then subjected to quality control by a designated officer. During the online follow-up intervention, follow-up nurses collected weekly data on the frequency and duration of patients’ online logins. Previous studies have established that patients achieving a completion rate of 90% or higher were classified as having excellent adherence, while those with a completion rate below 90% were considered to have poor adherence.

The functional status of elderly stroke patients was assessed using the FRAIL scale, developed by the International Society for Nutrition and Aging. This scale consists of five components: fatigue, resistance, ambulation, illness, and weight loss. Scores on the scale range from 0 to 5, with higher scores indicating greater frailty. A score of 0 represents the absence of frailty, scores of 1–2 indicate pre-frailty, and scores of 3–5 indicate frailty. The Cronbach’s *α* coefficient for this scale was calculated to be 0.826 (25).

The functional status of patients was evaluated using the Modified Barthel Index (MBI), which measures the ability to perform activities of daily living. The MBI consists of 10 components, and scores range from 0 to 100, with higher scores indicating higher proficiency. These scores are classified into four levels: 100 signifies no dysfunction, 61–99 indicates mild dysfunction, 41–60 indicates moderate dysfunction, and 0–40 indicates severe dysfunction. The internal consistency of the MBI, measured by the Cronbach’s *α* coefficient, exceeded 0.92 (26).

The health status of elderly stroke patients was assessed using the Short Form Health Survey (SF-36), which includes 36 items measuring various dimensions of health. These dimensions include physical function (PF), role physical (RP), bodily pain (BP), general health (GH), mental health (MH), vitality (VT), social function (SF), and role emotional (RE). A conversion formula was applied to each dimension to calculate a total score ranging from 0 to 100, which serves as an indicator of the patients’ quality of life. Higher scores indicate a better overall health status. The Cronbach’s *α* coefficient of the scale ranged from 0.7 to 0.9 (27).

To evaluate the compliance of Multicomponent Exercise Nursing Intervention in Elderly Stroke Patients with Frailty, we investigated the compliance in the intervention group. Researchers conducted weekly telephone follow-ups to collect data on the frequency and duration of patients’ exercise training sessions. Achieving 90% or more of the total prescribed exercise sessions during the intervention period was considered good compliance, while completing less was considered poor compliance.

### 2.6 Statistical analysis

Statistical analysis was performed using SPSS 22.0 software. Descriptive statistics were used to summarize measurement data, with normally distributed quantitative data presented as mean ± standard deviation and non-normally distributed data presented as median and interquartile range [M (P_25_, P_75_)]. Counting data were presented as frequencies and percentages. Intergroup comparisons were performed using independent sample *t*-tests or non-parametric rank sum tests, while comparisons among different time points were conducted using repeated measures ANOVA or generalized estimating equations. The chi-square test was used to compare rates between groups. Two-sided *p* values less than 0.05 were considered statistically significant.

### 2.7 Sample size

The sample size for this study was determined using the formula *n*_1_ = *n*_2_ = 2[(μ*_α_* + μ*_β_*)*σ*/*δ*]^2^, where pre-experiment data on frailty as the primary observation index indicated σ = 3.71 and δ = 2.64. With significance levels α = 0.05 and β = 0.10, the calculated sample size was *n*_1_ = *n*_2_ = 42 cases. To account for a 20% loss to follow-up, the final sample size was adjusted to 105 cases, which was ultimately increased to 125 cases in the actual study.

## 3 Results

A total of 125 elderly stroke patients were included in this study, with 63 cases in the intervention group and 62 cases in the control group. Table 2 shows that there was no statistically significant difference in the general information between the two groups (*p* > 0.05).

**Table 2:** Comparison of general data between two groups (*n* = 125).

Variable	Intervention group (*n* = 63)	Control group (*n* = 62)	*t*/χ^2^/*Z* value	*p* value
Age (years)	73.03 ± 7.53	73.50 ± 6.23	0.379 ^a^	0.706
Gender (*n*, %)			0.008 ^b^	0.927
Male	32 (50.8)	32 (51.6)		
Female	31 (49.2)	30 (48.4)		
Educational level (*n*, %)			5.708 ^b^	0.127
Primary and below	26 (41.3)	17 (27.4)		
Junior high school	13 (20.6)	24 (38.7)		
High school	19 (30.2)	15 (24.2)		
College and above	5 (7.9)	6 (9.7)		
Medical insurance			0.656 ^b^	0.418
Residents’ medical insurance	47 (74.6)	50 (80.6)		
City and town medical insurance	16 (25.4)	12 (19.4)		
Monthly income			0.884 ^b^	0.347
<3,000 yuan	43 (68.3)	47 (75.8)		
≥3,000 yuan	20 (31.7)	15 (24.2)		
Smoking (*n*, %)			0.068 ^b^	0.794
No	49 (77.8)	47 (75.8)		
Yes	14 (22.2)	15 (24.2)		
Alcohol drinking (*n*, %)			0.670 ^b^	0.413
No	56 (88.9)	52 (83.9)		
Yes	7 (11.1)	10 (16.1)		
Body mass index (BMI) (*n*, %)			3.640 ^b^	0.162
<18.5 (underweight)	2 (3.2)	3 (4.8)		
18.5–23.9 (normal weight)	32 (50.8)	28 (45.2)		
≥24.0 (overweight or obese)	29 (46.0)	31 (50.0)		
Hypertension (*n*, %)			0.729 ^b^	0.393
No	26 (41.3)	21 (33.9)		
Yes	37 (58.7)	41 (66.1)		
Diabetes mellitus (*n*, %)			1.993 ^b^	0.158
No	47 (74.6)	39 (62.9)		
Yes	16 (25.4)	23 (37.1)		
Heart disease (*n*, %)			0.169 ^b^	0.681
No	60 (95.2)	58 (93.5)		
Yes	3 (4.8)	4 (6.5)		
Degree of stroke			0.224 ^b^	0.636
Normal	10 (15.9)	8 (12.9)		
Mildly	53 (84.1)	54 (87.1)		
Systolic blood pressure (mmHg)	143.38 ± 24.64	142.19 ± 14.13	−0.330 ^a^	0.742
Diastolic blood pressure (mmHg)	74.75 ± 10.22	78.21 ± 12.03	1.736 ^a^	0.085
Leucocyte (109/L)	5.94 ± 2.57	6.50 ± 1.31	1.533 ^a^	0.129
Red blood cell (1,012/L)	4.31 ± 0.50	4.27 ± 0.47	−0.482 ^a^	0.631
Blood platelet (109/L)	175.33 ± 48.29	188.84 ± 78.06	1.165 ^a^	0.248
Hemoglobin (g/L)	127.35 ± 14.03	130.36 ± 14.76	1.167 ^a^	0.246
Fasting blood glucose (mmol/L)	5.66 ± 1.22	6.22 ± 2.31	1.665 ^a^	0.099
Total cholesterol (mmol/L)	4.21 ± 0.99	3.94 ± 1.02	−1.488 ^a^	0.139
Triglycerides (mmol/L)	1.33 (0.96,1.76)	1.47 (1.38,2.31)	0.588 ^c^	0.558

a *t value.*

b χ^2^ value.

c *Z value.*

Frailty scores were compared between exercise and control groups at pre-intervention, 4 weeks, and 12 weeks post-intervention. The exercise group showed a significant reduction in scores over time (Pre: 3.37 ± 0.63; 4 weeks: 2.43 ± 0.56; 12 weeks: 1.66 ± 0.87), while the control group’s scores remained stable (Pre: 3.33 ± 0.57; 4 weeks: 3.14 ± 0.43; 12 weeks: 3.15 ± 0.51). A repeated measures ANOVA showed significant effects of time and group on frailty scores, with a notable interaction indicating greater improvement in the exercise group (*F*_interaction_ = 47.824, *p* < 0.001, η^2^ = 0.388). Significant differences were also found between groups (*F*_group_ = 38.339, *p* < 0.001, η^2^ = 0.512) and over time (*F*_time_ = 76.225, *p* < 0.001, η^2^ = 0.505). *Post-hoc* pairwise comparisons revealed significant reductions in frailty scores in the exercise group at both 4 and 12 weeks post-intervention (*p* < 0.001), with further improvement at 12 weeks compared to 4 weeks (*p* < 0.05). The control group showed no significant changes over the same periods (Table 3).

**Table 3:** Pairwise comparison of Frail scale scores at different time points in the two groups (scores, $\bar{x}$ ± s).

Variable	*n*	Pre-intervention	4 weeks after intervention	12 weeks after intervention	*F* value	*p* value
Exercise group	63	3.37 ± 0.63	2.43 ± 0.56	1.66 ± 0.87	1.942	0.148
Control group	62	3.33 ± 0.57	3.14 ± 0.43^a^	3.15 ± 0.51^a^^,^^b^	98.104	<0.001***
*t* value		−0.350	7.882	11.629		
*p* value		0.727	<0.001***	<0.001***		

*F*_inter-group_ = 38.339, *p* < 0.001***, Eta-Squared (η^2^) = 0.512; *F*_time_ = 76.225, *p* < 0.001***, η^2^ = 0.505; *F*_interaction_ = 47.824, *p* < 0.001***, η^2^ = 0.388.

a Compared with pre-intervention, p < 0.05*….

b Compared with 4 weeks after intervention, p < 0.05*…

Table 4 shows the pairwise comparison of modified Barthel Scale scores over time for two groups. The exercise group (*n* = 63) had a significant score increase from pre-intervention [median (IQR): 85 (80, 85)] to 12 weeks post-intervention [90 (90, 95)], with improvements noted both compared to pre-intervention and 4 weeks post-intervention (*p* < 0.05). The control group (*n* = 62) showed no significant change from pre-intervention [82.5 (75, 90)] to 4 weeks [85 (80, 90)] and 12 weeks post-intervention [85 (80, 90)]. Statistical analysis showed notable differences between groups (Wald*χ^2^* = 34.397, *p* < 0.001, η^2^ = 0.049), over time (Wald*χ^2^* = 123.149, *p* < 0.001, η^2^ = 0.607), and in their interaction (Wald*χ^2^* = 42.380, *p* < 0.001, η^2^ = 0.361). *Post-hoc* tests revealed the exercise group scored significantly higher than the control group at 4 weeks (*z* = −2.335, *p* = 0.021) and 12 weeks (*z* = −5.084, *p* < 0.001) post-intervention.

**Table 4:** Pairwise comparison of modified Barthel scale score at different time points in the two groups [score, M (P_25_, P_75_)].

Variable	*n*	Pre-intervention	4 weeks after intervention	12 weeks after intervention	*F* value	*p* value
Exercise group	63	85 (80,85)	85 (80,90)^a^	90 (90,95)^a^^,^^b^	121.548	<0.001***
Control group	62	82.5 (75,90)	85 (80,90)^a^	85 (80,90)^a^	7.970	0.001***
*z* value		−0.023	−2.335	−5.084		
*p* value		0.981	0.021*	<0.001***		

Waldχ^2^_inter-group_ = 34.397, *p* < 0.001***, η^2^ = 0.049; Waldχ^2^_time_ = 123.149, *p* < 0.001***, η^2^ = 0.607; Waldχ^2^_interaction_ = 42.380, *p* < 0.001***, η^2^ = 0.361.

a Compared with pre-intervention, p < 0.05*….

b Compared with 4 weeks after intervention, p < 0.05*…

The results of the repeated measures ANOVA for the SF-36 scores in each dimension are shown in Table 5. Significant within-group changes were observed for Physical Functioning (PF), Role Physical (RP), Bodily Pain (BP), General Health (GH), Vitality (VT), Social Functioning (SF), Role Emotional (RE), and Mental Health (MH) dimensions (all *p* < 0.001), indicating improvements over time within both groups. However, the BP dimension did not show a significant inter-group difference (*F* = 0.265, *p* = 0.608). Notably, the interaction effect was significant for all dimensions except Role Emotional (RE) and Bodily Pain (BP) (*p* < 0.001 for PF, RP, GH, VT, SF, MH; *p* = 0.119 for RE; *p* = 0.001 for BP), suggesting that the changes over time differed significantly between the exercise and control groups in most dimensions. Specifically, the interaction effect was strongest for Vitality (VT) (*F* = 149.238, *p* < 0.001) and Social Functioning (SF) (*F* = 41.227, *p* < 0.001). The inter-group comparison also revealed significant differences for Physical Functioning (PF) (*F* = 12.722, *p* = 0.001), Role Physical (RP) (*F* = 15.165, *p* < 0.001), General Health (GH) (*F* = 62.253, *p* < 0.001), Vitality (VT) (*F* = 146.055, *p* < 0.001), Social Functioning (SF) (*F* = 8.092, *p* < 0.001), and Mental Health (MH) (*F* = 16.811, *p* < 0.001), while no significant difference was noted for Role Emotional (RE) (*F* = 0.008, *p* = 0.929).

**Table 5:** Results of ANOVA of repeated measurement of SF-36 scores in each dimension before and after intervention in two groups.

Variable	Within-group			Inter-group			Interaction		
	*F* value	*p* value	η^2^	*F* value	*p* value	η^2^	*F* value	*p* value	η^2^
PF	28.163	<0.001***	0.316	12.722	0.001***	0.094	29.884	<0.001***	0.329
RP	29.483	<0.001***	0.326	15.165	<0.001***	0.110	20.751	<0.001***	0.254
BP	17.487	<0.001***	0.223	0.265	0.608	0.002	5.995	0.001***	0.089
GH	125.490	<0.001***	0.673	62.253	<0.001***	0.336	118.760	<0.001***	0.661
VT	179.265	<0.001***	0.746	146.055	<0.001***	0.543	149.238	<0.001***	0.710
SF	191.262	<0.001***	0.758	8.092	<0.001***	0.062	41.227	<0.001***	0.403
RE	11.226	<0.001***	0.155	0.008	0.929	<0.001	2.165	0.119	0.034
MH	133.987	<0.001***	0.687	16.811	<0.001***	0.120	20.401	<0.001***	0.251

The pairwise comparison of SF-36 scores in each dimension at different time points for both the exercise and control groups is presented in Table 6. Significant within-group improvements were observed in the exercise group for Physical Functioning (PF), Role Physical (RP), General Health (GH), Vitality (VT), Social Functioning (SF), and Mental Health (MH) dimensions over time (all *p* < 0.001). For example, the PF score increased from a mean ± SD of 40.63 ± 7.85 at pre-intervention to 50.40 ± 12.19 at 12 weeks post-intervention. Similarly, the GH score improved significantly from 30.95 ± 11.21 to 59.52 ± 8.17 over the same period. In contrast, the control group showed no significant within-group changes in PF, RP, VT, SF, or MH dimensions (*p* > 0.05), although there was a slight improvement in GH scores which was not statistically significant (*p* = 0.865). The Role Emotional (RE) dimension did not exhibit significant changes over time in either group (*p* > 0.05). Significant inter-group differences were found for PF, RP, GH, VT, SF, and MH dimensions at 12 weeks post-intervention (all *p* < 0.001), indicating that the exercise group experienced greater improvements compared to the control group. For instance, the VT score in the exercise group increased from 25.48 ± 6.58 to 59.60 ± 11.26, whereas it only marginally increased from 25.32 ± 7.83 to 26.94 ± 10.65 in the control group. No significant inter-group differences were noted for RE (*p* > 0.05). The interaction effect was significant for PF, RP, GH, VT, SF, and MH (all *p* < 0.001), highlighting that the changes over time were more pronounced in the exercise group compared to the control group. However, the interaction for RE was not significant (*p* = 0.119).

**Table 6:** Pairwise comparison of SF-36 scores in each dimension at different time points in the two groups (scores, $\bar{x}$ ± s).

Variable	Pre-intervention	4 weeks after intervention	12 weeks after intervention	*F* value	*p* value
PF					
Exercise group	40.63 ± 7.85	43.81 ± 10.03^a^	50.40 ± 12.19^a^^,^^b^	46.925	<0.001***
Control group	40.64 ± 8.94	37.26 ± 9.61^a^	39.19 ± 11.35	11.406	<0.001***
*t* value	0.007	−3.728	−5.315		
*p* value	0.995	<0.001***	<0.001***		
RP					
Exercise group	23.02 ± 6.81	33.33 ± 15.55^a^	41.67 ± 22.90^a^^,^^b^	48.772	<0.001***
Control group	24.60 ± 3.18	27.02 ± 6.86	25.81 ± 4.45	1.837	0.164
*t* value	1.667	−2.945	−5.396		
*p* value	0.100	0.004**	<0.001***		
BP					
Exercise group	89.49 ± 13.37	90.35 ± 10.23^a^	83.42 ± 7.83^a^^,^^b^	21.521	<0.001***
Control group	90.97 ± 11.98	90.15 ± 10.77	88.85 ± 10.93	2.117	0.125
*t* value	0.651	−0.104	2.047		
*p* value	0.516	0.917	0.043*		
GH					
Exercise group	30.95 ± 11.21	52.22 ± 7.22^a^	59.52 ± 8.17^a^^,^^b^	246.071	<0.001***
Control group	32.10 ± 13.04	32.74 ± 13.23	32.58 ± 15.88	0.146	0.865
*t* value	0.526	−10.191	−11.899		
*p* value	0.600	<0.001***	<0.001***		
VT					
Exercise group	25.48 ± 6.58	41.75 ± 11.68^a^	59.60 ± 11.26^a^^,^^b^	330.317	<0.001***
Control group	25.32 ± 7.83	25.48 ± 9.04	26.94 ± 10.65	0.822	0.442
*t* value	−0.119	−8.713	−16.657		
*p* value	0.906	<0.001***	<0.001***		
SF					
Exercise group	38.80 ± 14.92	59.96 ± 10.28^a^	76.37 ± 9.67^a^^,^^b^	205.958	<0.001***
Control group	44.98 ± 21.63	51.08 ± 17.59^a^	58.06 ± 17.24^a^^,^^b^	27.955	<0.001***
*t* value	1.858	−3.442	−7.304		
*p* value	0.066	0.001***	<0.001***		
RE					
Exercise group	37.57 ± 11.19	42.33 ± 14.91^a^	47.09 ± 18.58^a^^,^^b^	11.688	<0.001***
Control group	40.32 ± 13.68	41.94 ± 14.70	44.09 ± 17.88	1.783	0.173
*t* value	1.232	−0.148	−0.921		
*p* value	0.220	0.882	0.359		
MH					
Exercise group	40.89 ± 9.21	45.21 ± 10.67^a^	54.54 ± 10.69^a^^,^^b^	128.637	<0.001***
Control group	38.93 ± 9.71	41.10 ± 7.50^a^	44.77 ± 7.02^a^^,^^b^	26.568	<0.001***
*t* value	−1.164	−2.494	−6.048		
*p* value	0.062	0.014*	<0.001***		

a Compared with pre-intervention, p < 0.05*….

b Compared with 4 weeks after intervention, p < 0.05*..

Following a 4-week intervention, 51 individuals (80.9%) in the intervention group demonstrated satisfactory compliance. This number decreased to 48 individuals (76.2%) with satisfactory compliance after a 12-week intervention period. No falls, muscle injuries, or joint problems were reported in the intervention group throughout the intervention duration.

## 4 Discussion

To the best of our knowledge, this study is the first randomized controlled trial to implement a multicomponent exercise nursing model in elderly stroke patients with frailty. The trial compares the effects of a multicomponent exercise nursing model with a standard nursing approach on frail elderly stroke patients, focusing on factors such as frailty, activities of daily living, and quality of life. The findings indicate that the multicomponent exercise nursing intervention is effective in improving the frailty status of elderly stroke patients. Furthermore, significant improvements were observed in both functional capacity for activities of daily living and overall quality of life.

Stroke patients are at an increased risk of developing frailty due to advanced age and the sudden onset of the disease. Frailty, in turn, serves as an independent risk factor for cardiovascular and cerebrovascular diseases, creating a cyclical relationship. Research shows that stroke patients with frailty experience a lower health-related quality of life and a more pronounced decline in health compared to non-frail patients (6). Consistent with the findings of this study, multicomponent exercise has been shown to be effective in improving frailty in older patients hospitalized for heart failure (28). Multicomponent exercise, which combines various exercises, has been shown to enhance cardiopulmonary function, increase muscle strength, flexibility, and coordination, reduce the risk of falls, and promote mental well-being. Its benefits extend to conditions such as Alzheimer’s disease (29, 30), breast cancer (31), hypertension (32), and diabetes (20). The endorsement of multicomponent exercise by the World Health Organization (WHO) as a tool for developing personalized exercise regimens underscores its effectiveness and importance (33).

Individuals with stroke are at risk of experiencing a decline in activities of daily living, which can have long-term consequences if not addressed promptly. For elderly individuals, participating in comprehensive exercise programs is crucial for restoring independence in daily life activities. Achieving independence not only reduces the burden on caregivers but also enhances overall well-being. A randomized controlled trial conducted among frail elderly individuals in the community demonstrated that participation in a multicomponent exercise regimen effectively improves ADL (34), which aligns with the findings of the present study. Frailty has been shown to lead to decreased endurance, muscle strength, balance function, gait, and body flexibility in patients. Multicomponent exercise training combines the features and benefits of different training methods, thereby enhancing overall body function in frail patients and ultimately improving their frailty status (35).

The multidimensional concept of quality of life includes aspects such as physical health, psychological well-being, social connections, and environmental satisfaction. This research illustrates that a multicomponent exercise nursing intervention can enhance patients’ quality of life, which aligns with findings from previous studies (36). Physical exercise has the potential to improve physical health, facilitate functional recovery, stimulate the release of endorphins (37), alleviate anxiety and depression (38), and enhance self-esteem and self-assurance among patients. Physical exercise has also been shown to improve social interactions and foster a sense of belonging and participation within a community (39). Moreover, Watson’s humanistic care theory emphasizes the importance of addressing individual patient needs and values, as well as monitoring psychological and emotional well-being. By incorporating the principles of humanistic care into a multicomponent exercise nursing intervention, the overall quality of life for patients can be significantly enhanced.

The high adherence observed in our study is consistent with findings from a previous multicenter randomized controlled trial (40). This may be attributed to the integration of the PRECEDE-PROCEED model and humanistic care model in our study, as well as the utilization of nursing theory to enhance patient motivation (41). The incorporation of the PRECEDE-PROCEED model has been shown to effectively guide the planning, implementation, and evaluation of health promotion programs. This model provides a systematic framework for planning and evaluating health behavior change interventions through the use of flowcharts (22). Unlike traditional approaches where patients passively receive education from nurses, this model emphasizes empowering patients to take an active role in their own healthcare decisions, promoting sustained compliance and improved health outcomes. Numerous studies have demonstrated the successful application of this theory to diseases such as diabetes (42, 43), hypertension (44, 45), and obesity (46, 47). Watson’s humanistic care theory emphasizes the significance of humanistic care and the importance of showing respect for individuals within the nursing profession. At the core of Watson’s theory is the idea that nursing is a moral and philosophical endeavor aimed at enhancing the well-being of individuals, families, and communities (23). Nurses are responsible for assisting patients in achieving physical, mental, emotional, and spiritual equilibrium through compassionate interactions (48). The application of this theory to primipara (49), dementia patients (50), and end-stage patients (51) has been shown to enhance patient quality of life and satisfaction. This study integrates these two nursing theories into the intervention program, resulting in improved patient compliance and enhanced implementation effectiveness.

Throughout the course of this study, no adverse events such as falls or muscle and joint injuries occurred, indicating the program’s strong emphasis on safety and scientific rigor. The program framework was developed based on expert consensus, guidelines, and the specific physical and psychological needs of elderly stroke patients, utilizing the PRECEDE-PROCEED model (52) and humanistic care model. The exercise content was tailored to better suit the frailty of elderly stroke patients. After the initial draft, a meeting was held with a panel of experts from various clinical specialties, including neurology, clinical nursing, stroke rehabilitation, nursing management, and psychotherapy, all with extensive clinical experience. Based on feedback from preliminary experimental results, adjustments were made to the intensity and content of the intervention, following the principles of gradual and individualized intervention measures. The finalized version of the intervention program for elderly individuals with stroke was formulated. Therefore, the intervention program of this study is scientifically sound to some extent.

This study has certain limitations. Firstly, the randomized controlled trial had a duration of only 3 months, requiring further long-term follow-up to assess the intervention’s lasting effects. Secondly, the patient sample was limited to a Class iii Grade A hospital in Wuxi City, potentially limiting its generalizability. Future research should involve multi-center, large-sample trials to confirm the feasibility and effectiveness of the intervention. In addition, the control group in this study received standard care instead of a targeted exercise intervention. It is recommended that future research compare the multicomponent exercise intervention with a specific exercise intervention to further validate the effectiveness of the former. Furthermore, to ensure the safety of the trial, this study exclusively included patients with a muscle strength of at least grade 4. This criterion inherently limited the generalizability of the study’s findings. Additionally, the study did not account for the lesion location or the affected hemisphere, factors which could potentially influence the outcomes. Future research should aim to broaden the inclusion criteria and conduct a more nuanced analysis of patients with varying conditions to enhance the generalizability and accuracy of the results.

## 5 Conclusion

The findings of the present study suggest that among elderly stroke patients with frailty, a multicomponent exercise nursing intervention is safe, feasible, and has the potential to significantly improve patients’ frailty status, activities of daily living, and quality of life compared to routine nursing care. Future research should consider including additional evaluation measures to provide a more comprehensive assessment of the impact of a multicomponent exercise nursing intervention in this population.

## Data Availability

The raw data supporting the conclusions of this article will be made available by the authors, without undue reservation.
